# Leptin differentially regulates chondrogenesis in mouse vertebral and tibial growth plates

**DOI:** 10.1186/s12891-017-1601-6

**Published:** 2017-05-31

**Authors:** Bo Yu, Kaibiao Jiang, Bin Chen, Hantao Wang, Xinfeng Li, Zude Liu

**Affiliations:** Department of Spine Surgery, Ren Ji Hospital, School of Medicine, Shanghai Jiao Tong University, 160 Pujian Rd, Shanghai, 200127 China

**Keywords:** Leptin, Chondrogenesis, Vertebral growth plate, Tibial growth plate, Primary cell culture, Chondrocyte

## Abstract

**Background:**

Leptin plays an important role in mediating chondrogenesis of limb growth plate. Previous studies suggest that bone structures and development of spine and limb are different. The expression of Ob-Rb, the gene that encodes leptin receptors, is vertebral and appendicular region-specific, suggesting the regulation of leptin on VGP and TGP chondrogenesis may be very different. The aim of the present study was to investigate the differential regulation of leptin on the chondrogenesis of vertebral growth plate (VGP) and tibial growth plate (TGP).

**Methods:**

We compared the VGP and TGP from wild type (C57BL/6) and leptin-deficient (ob/ob) mice. We then generated primary cultures of TGP and VGP chondrocytes. By treating the primary cells with different concentrations of leptin in vitro, we analyzed proliferation and apoptosis of the primary chondrocytes from TGP and VGP. We further measured expression of chondrogenic-related genes in these cells that had been incubated with different doses of leptin.

**Results:**

Leptin-deficient mice of 8-week-old had shorter tibial and longer vertebral lengths than the wide type mice. Disturbed columnar structure was observed for TGP but not for VGP. In primary chondrocyte cultures, leptin inhibited VGP chondrocyte proliferation but promoted their apoptosis. Collagen IIA and aggrecan mRNA, and the protein levels of proliferation- and chondrogenesis-related markers, including PCNA, Sox9, and Smad4, were downregulated by leptin in a dose-dependent manner. In contrast, leptin stimulated the proliferation and chondrogenic differentiation of TGP chondrocytes at physiological levels (i.e., 10 and 50 ng/mL) but not at high levels (i.e., 100 and 1000 ng/mL).

**Conclusion:**

Leptin exerts a stimulatory effect on the proliferation and chondrogenic differentiation of the long bone growth plate but an inhibitory effect on the spine growth plate. The ongoing study will shed light on the regulatory mechanisms of leptin in bone development and metabolism.

## Background

Longitudinal bone growth is mainly attributed to the endochondral ossification process that occurs in the epiphyseal growth plates [1, 2]. Endochondral ossification is a tightly regulated process where the chondrocytes in the growth plate undergo a series of orchestrated events including proliferation, maturation, hypertrophy, apoptosis, and mineralization by matrix vesicles, ultimately leading to replacement of cartilage with bone [3–5]. Growth plate chondrogenesis is subject to regulations of multiple autocrine or paracrine factors such as bone morphogenetic proteins, parathyroid hormone-related protein, Indian hedgehog, growth hormone, sex steroids, and leptin [3, 5–8]. Leptin, encoded by the obese gene, is a circulating hormone secreted primarily from adipocytes. Leptin can potentiate bone growth in ob/ob mouse and promote differentiation of cultured growth plate chondrocytes, suggesting its critical role in the regulation of chondrogenesis in the growth plate [9–11].

Our previous work suggested that the active form of the leptin receptor (Ob-Rb) was expressed differentially in the tibial and spinal growth plates. The region-specific expression patterns of Ob-Rb gene were associated with mouse development, and were more prominent around puberty [12, 13]. Consistent with the observation, a previous study indicated that the effects of leptin on bone differed significantly between axial and appendicular regions, with ob/ob mice having significantly increased vertebral length, lumbar bone mineral content (BMC), lumbar bone mineral density (BMD), yet shorter femora and lower limb BMC and limb BMD [14]. Furthermore, abnormal leptin level was associated with defective vertebral development but not limb development in children with adolescent idiopathic scoliosis [15]. Therefore, the regulation of leptin on the chondrogenesis of the growth plate may be region-specific: its effect on vertebral growth plate (VGP) may be distinct from that on tibial growth plate (TGP).

To date, the region-specific regulation of leptin on the chondrogenesis of the growth plate still awaits demonstration. A previous study has suggested that leptin may function differently in femur and lumbar bones. In this study, leptin-deficiency was shown to inhibit deposition of mineral and matrix in femurs but no difference was observed for lumbar vertebrae [16]. Therefore, it is conceivable that chondrogenesis of VGP and TGP may be subject to modulation by different mechanisms. Given the previous observations [12, 14, 15], we hypothesize that leptin differentially regulates chondrogenesis of growth plates in vertebral and appendicular regions. Clarifying the region-specific regulation of leptin on chondrogenesis of growth plates may help us better understand the key roles of leptin in the regulation of bone development, and is closely related to the pathogenesis of abnormalities in bone development and bone metabolism [17, 18].

In this study, we isolated and established primary cultures for the TGP and VGP chondrocytes from neonatal wild type mice. Using these primary cell cultures, we tested the hypothesis that leptin differentially regulates chondrogenesis in the TGP and in the VGP. By treating the primary chondrocytes with different concentrations of leptin, we systematically characterized chondrogenic-related features of these cells at cellular and molecular levels. Specifically, we evaluated cell proliferation and apoptosis in the TGP and VGP primary chondrocytes, which are key cellular events in the growth plate chondrogenesis. We further measured the dynamics of the chondrogenic-associated molecular markers upon the treatment with different doses of leptin. We could demonstrate that leptin exerted opposite effects on chondrogenesis in the VGP and in the TGP.

## Methods

### Animals

C57BL/6 and ob/ob mice were purchased from Shanghai Laboratory Animal Co. Ltd. (SLAC, Shanghai, China), and maintained regularly with food and water readily accessible.

### Primary cell culture of growth plate chondrocytes

Vertebral and tibial growth plates were removed sterilely from neonatal C57BL/6 mice and finely minced in PBS. The minced tissues were transferred to a 50 mL falcon tube and washed by PBS for three times. The tissues were digested in DMEM/Ham’s F12 medium containing 0.1% of Trypsin and 0.1% EDTA at 37 °C for 30 min. Chondrocytes were isolated by collagenase digestion at 37 °C for overnight, for which 0.2% of collagenase was made in DMEM/Ham’s F12 medium supplemented with 5% fetal bovine serum. Upon isolation, chondrocytes were resuspended and cultured in DMEM/Ham’s F12 supplemented with 100 U/mL penicillin, 100 μg/mL streptomycin, and 10% fetal bovine serum. The cultures were incubated at 37 °C in a humidified atmosphere of 5% CO_2_/95% O_2_.

### Leptin treatment

Primary chondrocytes were seed in 6-well plate at a density of 1–2 × 10^5^ cells/well. When cells were grown to 70 to 80% confluency, the cell cultures were continued to incubate in serum-free medium for overnight until leptin treatment, where different concentrations of leptin were included (0, 10, 50, 100, and 1000 ng/mL) to treat the cells for 48 h. Recombinant mouse leptin (ProSpec bio) was used in all experiments.

### Cell proliferation assay

Cell proliferation assay (CCK-8 assay) was based on reduction of a tetrazolium salt into a water-soluble formazan dye in a living cell. The assay was performed according to the manufacturer’s manual (Cell Counting Kit-8, Dojindo Molecular Technologies, Inc., USA). Absorbance at 450 nm was recorded at end time points.

### RNA extraction and quantitative real time-PCR

The leptin-treated cells were subject to RNA extraction by Trizol reagent (Invitrogen) according to the manufacturer’s manual. cDNA synthesis was performed by One-Step TaKaRa PrimescriptTM RT Reagent Kit (TaKaRa, Japan). Gene expression of collagen II, Aggrecan, and cyclin D1 was measured by real-time PCR performed with SYBR Premix Ex Taq (TaKaRa, Japan) in a LightCycler (Roche Diagnostics) according to the manufacturer’s protocol. Real time PCR was performed with 20 amplification cycles including a step of 95 °C for 5 s and a step of 60 °C for 20 s. The relative mRNA fold change was expressed as 2^-ΔΔCt^ using GAPDH as the reference gene. The forward and reverse primer sequences of the genes tested are listed as the following: Col2a1 (collagen type II alpha 1): 5′-GGTGGAGCAGCAAGAGCAA-3′ and 5′-CGTCGCCGTAGCTGAAGTG-3′; Acan (aggrecan): 5′-CTAGCTGCTTAGCAGGGATAACG-3′ and 5′-CCGCAGAGTCACAAAGACCAA-3′; Cyclin D1: 5′-CCCACGATTTCATCGAACACT-3′ and 5′-GTGCATGTTTGCGGATGATC-3′; and GAPDH: 5′-CATCCGTAAAGACCTCTATGCCAAC-3′ and 5′-ATGGAGCCACCGATCCACA-3′.

### Western blot

Western blot was performed for the whole cell lysates from the leptin-treated cells. Anti-collagen-IIa and anti-leptin receptor antibodies were purchased from Abcam. Anti-proliferating cell nuclear antigen (PCNA), anti-Sox9, anti-Smad4, and anti-GAPDH antibodies were purchased from Cell Signaling Technology (Beverly, MA, USA). All secondary antibodies were purchased from Jackson ImmunoResearch (West Grove, PA).

### Immunofluorescence microscopy

For immunofluorescent microscopy, cells were fixed with 4% paraformaldehyde for 15–20 min. Following wash by PBS, 0.1% Triton/PBS was added and kept for 15 min. Blocking was done by incubation with 5% BSA/PBS for 25 min. Mixture of desmina-SMA (diluted at 1:100 by PBS) were added after remove of BSA/PBS and kept overnight at 4 °C. After wash with PBS, FITC-Cy3 (diluted at 1:100 by PBS) was added to stain the cells for 2–4 h, followed by washing in PBS for 3 times. Then 5 μg/mL Dako dye was added and kept for 5 min. After addition of antifade mounting medium, images were recorded by a confocal laser scanning microscope.

### Cell cycle analysis

After incubated with leptin for 48 h, cells were collected by centrifugation at 1000 rpm and resuspended in 1 mL of cold PBS. Cells were fixed by cold 10% ethanol at 4 °C for at least 2 h. After fixation, the cells were then washed by PBS and stained by propidium iodide (PI) (final concentration 100 μg/mL in PBS) at 37 °C for 30 min. Flow cytometry was performed using FL 488 nm.

### Analysis of apoptosis

Annexin V-PI staining was applied to test apoptosis of leptin-treated chondrocytes. Briefly, treated cells were harvested and washed by cold PBS. An Annexin buffer solution was used to resuspend the cells. A mixture of PI (final concentration 100 μg/mL) and Annexin V-Alexa Fluor488 conjugate was then added to stain the cells at room temperature. Flow cytometry was used to analyze apoptosis with the parameters of 494/518 nm set for Annexin V dectection and 535/617 nm for PI.

### Toluidine blue staining

Tibial and vertebral growth plates were fixed in 10% formalin, embedded in glycol methacrylate, and sliced into 4-μm sections. Toluidine blue staining was performed to visualize the morphology of chondrocytes. The length of the proliferative zone was measured using Win-ROOF software (Mitani-Corp, Fukui, Japan).

### Statistical analysis

Measurements of lengths and mRNA expression levels were expressed as mean ± SD. For comparisons between two means, Student’s *t*-test was performed with a *P*-value < 0.05 considered statistically significant. Statistical analysis was performed using the SPSS (SPSS Inc., USA) statistical package.

## Results

### Distinct VGP and TGP morphology in leptin-deficient mice suggests differential regulation of leptin in VGP and TGP chondrogenic differentiation

**Fig. 1:**
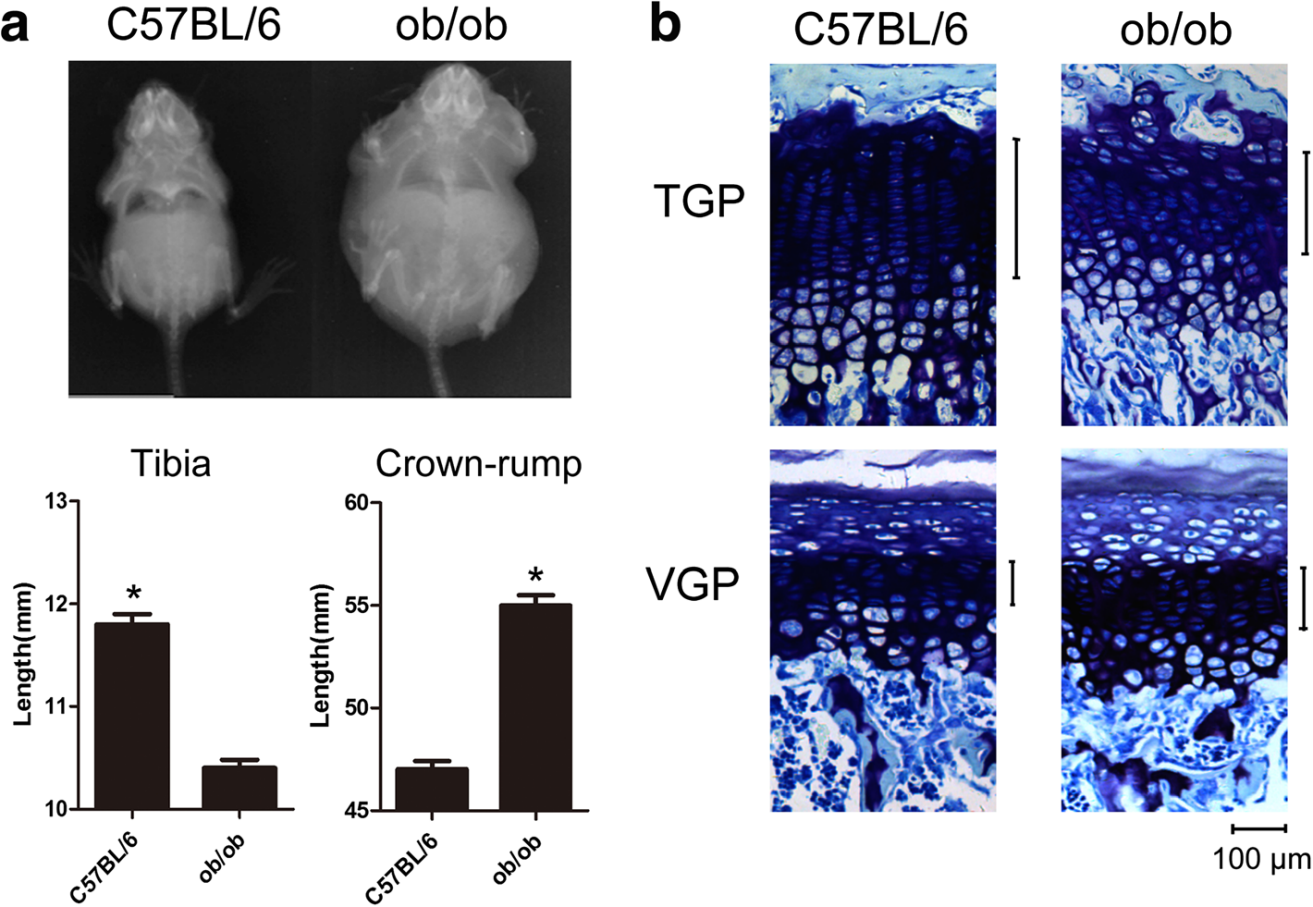
Distinct VGP and TGP morphology in leptin-deficient mice suggests differential regulation of leptin in VGP and TGP chondrogenic differentiation. **a** Plan radiography showing tibial and crown rump lengths of wild type C57BL/6 mouse (left) and ob/ob mouse (right). Measurement of the lengths is shown in the bottom panel. (* *p* < 0.05, Student’s *t*-test, *n* = 3) (**b**) Toluidine blue staining for TGP and VGP of wild type and ob/ob mice. The bracket indicates the lengths of the proliferative zones in VGP and TGP of different mice. Scale bar, 100 μm

In order to evaluate the potential effect of leptin on the proliferation and chondrogenic differentiation of VGP and TGP, we first measured and compared the crown-rump and tibial lengths of the wild type C57BL/6 mice with those of the ob/ob mice. Plan radiography results showed that ob/ob mouse of 8-week-old had significantly shorter tibial and longer crown-rump lengths compared with the C57BL/6 mice (Fig. 1a). We further performed histomorphometry for VGP and TGP that had been stained by toluidine blue. Strikingly, ob/ob mouse showed disarrayed columnar structure in TGP, for which such phenotype was not observed for VGP (Fig. 1b). Since ob/ob mouse is recessive for the mutated leptin gene, the observation suggests that leptin deficiency may have resulted in the disturbed columnar structure of TGP. Additionally, it appeared that for ob/ob mouse, the proportion of the proliferative zone in the growth plate was greater in VGP than in TGP (Fig. 1b). The proliferative zone is defined as the zone occupied by flattened chondrocytes that are aligned in longitudinal columns [19]. Taken together, the differential TGP and VGP morphology and histomorphometry in wild type and ob/ob mice are indicative of the potentially differential regulation of leptin in VGP and TGP chondrogenic differentiation.

### Primary cell cultures of TGP and VGP chondrocytes maintained chondrogenic-specific characteristics

**Fig. 2:**
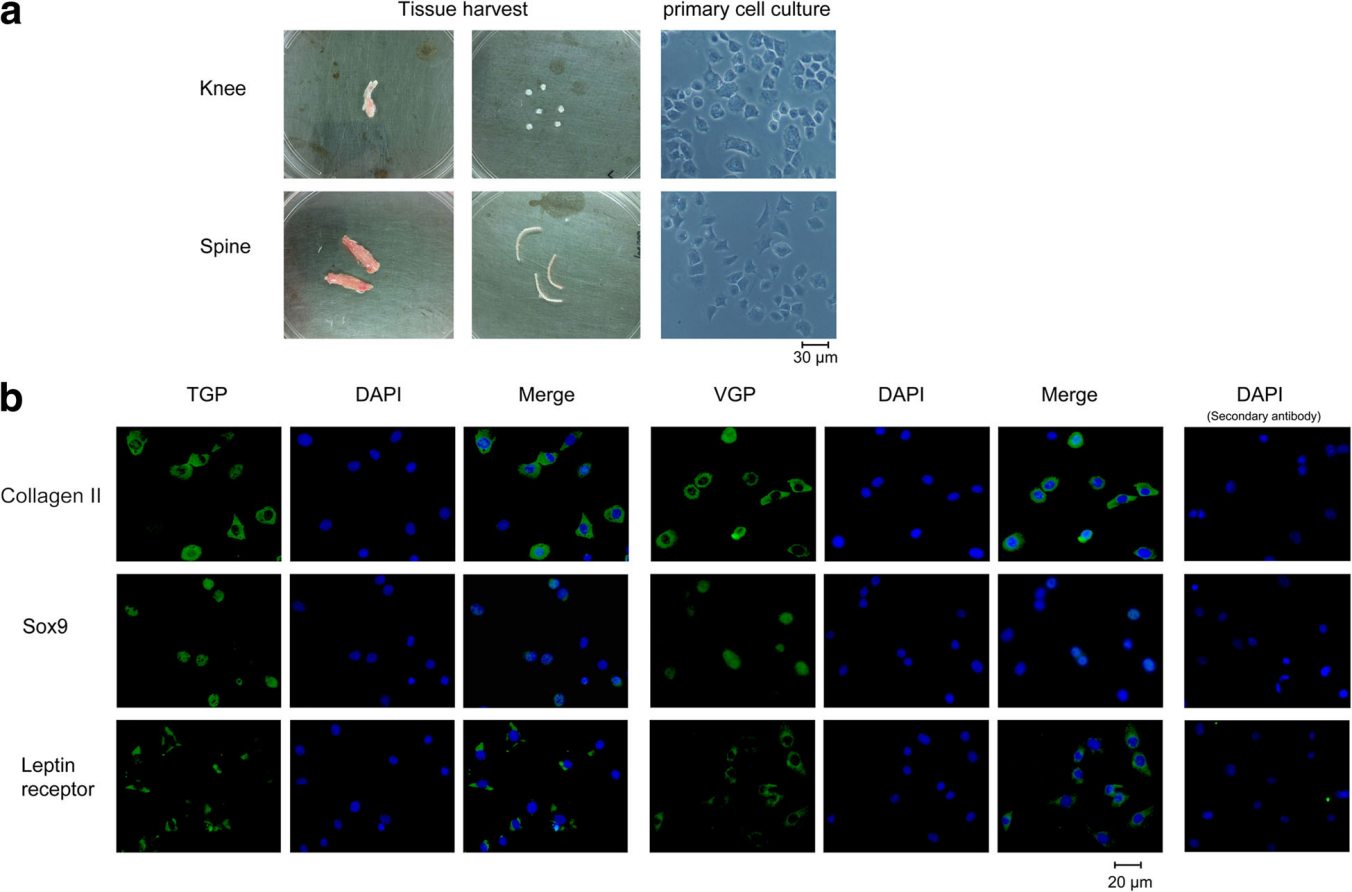
Primary cell cultures of TGP and VGP chondrocytes maintained the chondrogenic characteristics. **a** Bright field images of the dissected TGP and VGP from neonatal wild type mice (left and middle panels). The right panels show the microscopic images of the primary chondrocytes. Scale bar, 30 μm. **b** Immunofluorescent microscopic images showing TGP and VGP primary chondrocytes stained for collagen IIA, Sox 9, and leptin receptor. The right most panels represent the control images obtained by using only the secondary antibodies for the immunofluorescent imaging of Collagen II, Sox9, and leptin receptor, respectively

To better understand the regulation of leptin on chondrocyte proliferation and differentiation in TGP and VGP, we isolated chondrocytes from these two sites in neonatal wild type C57BL/6 mice for primary culture. The TGP and VGP tissues were aseptically removed and subject to isolation by collagenase digestion (Fig. 2a). Phase-contrast microscopy showed the cultured chondrocytes were mainly polygonal in morphology, with round or oval nuclei inside. Immunofluorescence microscopy further confirmed that both VGP and TGP chondrocytes expressed chondrogenic-specific makers and were stained positive for collagen II, Sox9, and leptin receptors, whereas control experiments using only secondary antibodies did not show non-specific signals (Fig. 2b). These results suggest that during the primary culture of TGP and VGP chondrocytes has maintained their chondrogenic characteristics, and is suitable for in vitro characterization of the functions of leptin on TGP and VGP proliferation and differentiation.

### Leptin inhibited chondrocyte proliferation and stimulated apoptosis in VGP

**Fig. 3:**
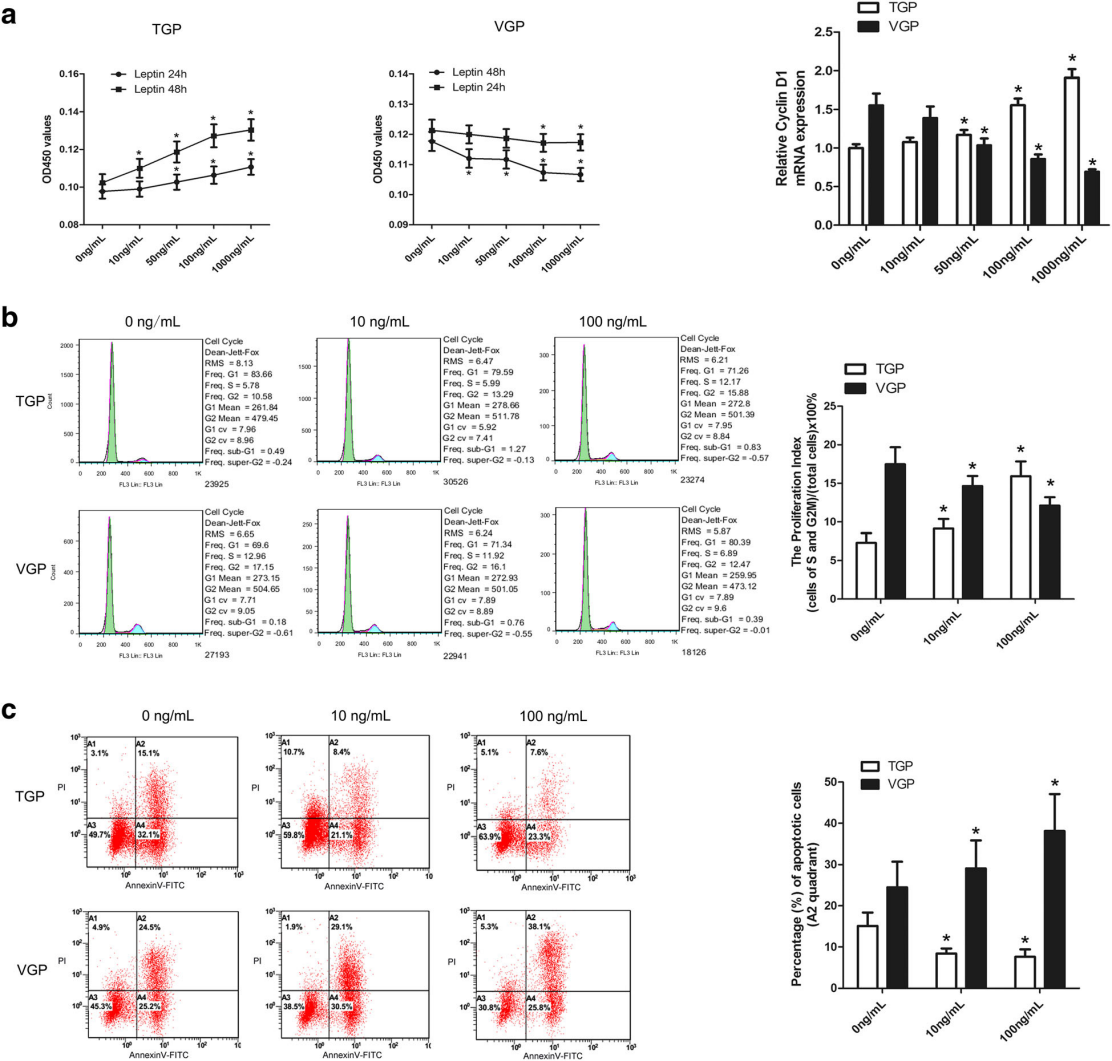
Leptin inhibited chondrocyte proliferation and stimulated apoptosis in VGP. **a** Colorimetric based cell proliferation assay (CCK-8 assay) for primary TGP and VGP chondrocytes that had been treated with recombinant leptin for 24 h or 48 h at different concentrations (0, 10, 50, 100, and 1000 ng/mL). Right panel: cyclin D1 mRNA level was measured by real time quantitative PCR for the leptin-treated cells using GAPDH as the reference gene. (* *p* < 0.05, Student’s *t*-test, *n* = 3). **b** Cell cycle analysis of the TGP and VGP chondrocytes treated with different concentrations of leptin (0, 10, and 100 ng/mL). Right panel: quantification of the proliferation index that is defined by the faction of S and G2/M cells in the total cells (%). (* *p* < 0.05, Student’s *t*-test, *n* = 3). **c** Flow cytometry analysis of apoptosis for the primary TGP and VGP chondrocytes treated with 0, 10, and 100 ng/mL of leptin. Right panel: quantification of the apoptotic cell populations (A2) from the flow cytometry analysis of apoptosis (* *p* < 0.05, Student’s *t*-test, *n* = 3)

In order to determine the effect of leptin on the proliferation of the TGP and VGP chondrocytes, we incubated the primary chondrocytes with various concentrations (i.e., 0, 10, 50, 100, and 1000 ng/mL) of recombinant mouse leptin for either 24 h or 48 h. For TGP, we found that leptin could stimulate the growth of chondrocytes in a dose-dependent manner, and at a given concentration of leptin, extended 48 h-incubation conferred faster cell proliferation than 24 h-incubation (Fig. 3a, left panel). Interestingly, the pattern of cell proliferation was reversed for VGP chondrocyte primary cells when incubated with leptin. Cell proliferation had been stagnant during the incubation course, and higher concentrations of leptin imposed slower proliferation. At a given concentration of leptin, prolonged incubation of 48 h could result in more significant inhibitory effect than short incubation of 24 h (Fig. 3a, middle panel). Quantitative real time PCR measurement of the expression level of cyclin D1 further confirmed this notion (Fig. 3a, right panel). These data suggest that in TGP leptin may stimulate chondrocyte growth while in VGP it may inhibit chondrocyte growth.

To confirm the opposite effects of leptin in TGP vs. VGP, we performed cell cycle analysis to quantify the proliferation index (i.e. the fraction of S and G2/M phase cells among total cells). Indeed, when TGP chondrocytes were treated with leptin for 48 h, increasing proliferation index was observed for elevated leptin concentrations. In contrast, the proliferation index has been constantly decreased with increasing level of leptin, further confirming the inhibitory effect of leptin on VGP chondrocytes (Fig. 3b).

Since chondrocytes in growth plate constantly undergo proliferation and differentiation, and mainly account for the bone development, we were wondering if inhibition of leptin on VGP chondrocyte growth has a biological implication in chondrocyte differentiation. Given that apoptosis plays an important role in cell differentiation and tissue remodeling, we further investigated if leptin could influence apoptosis of TGP and VGP chondrocytes. Flow cytometry analysis of TGP and VGP chondrocytes that had been incubated with increasing concentrations of leptin (i.e., 0, 10, and 100 ng/mL) revealed that leptin may have promoted apoptosis in VGP chondrocytes, while apoptosis in TGP chondrocytes was decreased with increasing dose of leptin treatment (Fig. 3c). Taken together, analyses of cell proliferation and apoptosis suggest that opposite regulation modes of leptin exist in two distinct anatomical structures of TGP and VGP: in TGP, leptin stimulates cell proliferation and inhibits apoptosis, while the effects of leptin are reserved in VGP.

### Leptin inhibited chondrogenesis in VGP but not in TGP

**Fig. 4:**
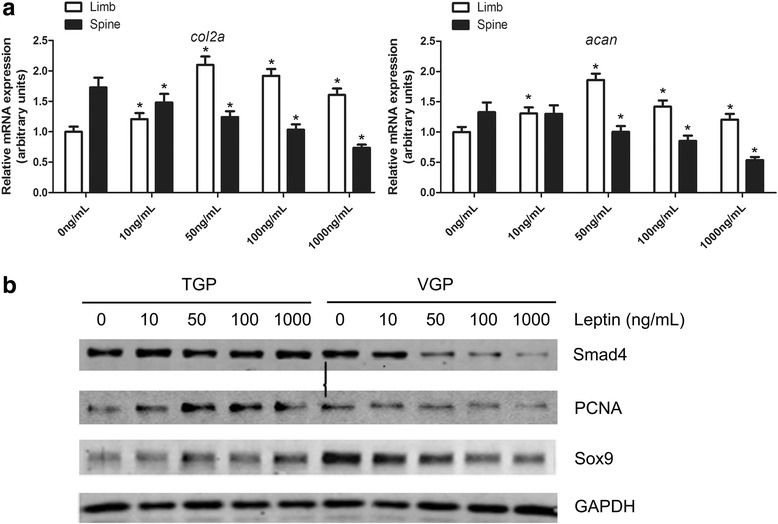
Leptin inhibited chondrogenesis in VGP but not in TGP. **a** Gene expression levels of collagen IIA (*col2a*) and aggrecan (*acan*) were measured by real time quantitative PCR for the primary cells treated with 0, 10, 50, 100, and 1000 ng/mL of leptin. GAPDH was used as the reference gene. (* *p* < 0.05, Student’s *t*-test, *n* = 3). **b** Proteins levels of chondrogenic-related markers, including Smad4, PCNA, and Sox9, were assayed by Western blotting. The primary TGP and VGP chondrocytes were treated with 0, 10, 50, 100, and 1000 ng/mL of leptinok

To illustrate the mechanism of differential regulation of leptin at the molecular level, we evaluated the expressions of multiple chondrogenic-specific markers in VGP and TGP chondrocytes. We first evaluated the mRNA levels of *col2a* and *acan*, which encode collagen IIA and aggrecan, respectively, in the TGP and VGP primary chondrocytes that were incubated with various concentrations of leptin (0, 10, 50, 100, and 1000 ng/mL). Collagen type II is the characteristic fibrillary collagen of cartilage and subject to developmentally regulations. Less differentiated chondroprogenitor cells mainly express collagen IIA [20, 21]. Aggrecan is a marker of differentiated chondrocytes [4]. Interestingly, in VGP chondrocytes, both *col2a* and *acan* mRNA levels were downregulated by leptin in a dose-dependent manner, suggesting leptin generally inhibits chondrogenesis in VGP (Fig. 4a). Such inhibition of chondrogenesis was not observed for TGP chondrocytes incubated with leptin, further indicating the opposite roles of leptin in VGP and TGP chondrogenesis (Fig. 4a).

Substantiating this notion, we further observed that the protein levels of multiple chondrogenic markers, including Sox9 and Smad4, were downregulated in VGP primary cells that were incubated with leptin. The effects also appeared to be dependent on the dosage of leptin (Fig. 4b). On a side note, PCNA was an important cell proliferation marker. Sox9 is an early chondrogenic transcription factor that is required for regulating successive differentiation steps in chondrocytes [22, 23]. Smad4 is a crucial downstream effector in the proliferation and differentiation of chondrocytes. This result confirms that leptin may inhibit chondrogenesis in VGP but not in TGP in general.

On the other hand, we also observed that the promotive role of leptin on TGP chondrocytes reached the maximum at around 50 ng/mL (Fig. 4a, open bars). At either lower or higher concentrations than 50 ng/mL, the effect of leptin diminished symmetrically, probably suggesting the action of leptin on the normal TGP chondrogenesis is restricted by the number of the leptin receptors (see Discussion) on the chondrocytes.

## Discussion

In this study, we confirmed that leptin differentially regulates chondrogenesis in primary TGP and VGP chondrocyte cultures. We isolated the chondrocytes from these two sites in neonatal wild type mice, and showed that the primary cell cultures of TGP and VGP chondrocytes maintained the chondrogenic-specific features as judged by cell morphology and the expression of chondrogenic markers including collagen II, Sox9, and leptin receptors. At the organ level, plan radiography showed that ob/ob mouse of 8-week-old had significantly shorter tibial and longer crown-rump lengths than the wild type mice. At the cellular level, leptin-deficient (ob/ob) mouse showed disarrayed columnar structure in TGP but not in VGP. On the other hand, cell cycle analysis by flow cytometry further confirmed that in VGP chondrocytes that had been incubated with exogenous leptin, cell proliferation was inhibited, yet apoptosis was enhanced. At the molecular level, exogenous leptin repressed the levels of chrodrogenic-specific markers, including collagen IIA, aggrecan, Sox9, and Smad4 in VGP, and the effects were leptin dose-dependent. Therefore, leptin imposes opposite effects on the chondrogenesis of primary TGP and VGP chondrocytes, with a chondrogenesis-inhibitory effect being dominant in VGP but not in TGP.

Consistent with a previous finding, where ob/ob mice at 6 months of age had shorter femora but longer vertebral lengths than the wide type mice [14], we found that ob/ob mouse of 8-week-old had significantly shorter tibial and longer crown-rump lengths compared with the wild type mice. Therefore, given the mouse age at the time of measurement, we postulate that the effect of leptin on longitudinal bone growth may last for at least a period of time window. However, it is worth determining the span of this temporal window when leptin maintains its function on chondrogenesis since it is a tightly regulated process for bone growth. On the other hand, the disrupted columnar structure of TGP displayed by toluidine blue staining in the leptin-deficient mice can at least partially account for the defective tibial development in the ob/ob mice. Strikingly, in the VGP of the ob/ob mice, such a columnar structural disruption was not observed, suggesting a very different role of leptin at these two distinct anatomical sites during chondrogenesis.

The distinct effects of leptin on vertebral and appendicular bones have been suggested in a leptin-deficient (ob/ob) mouse model: Ealey et al. [16] reported that leptin-deficiency could inhibit matrix mineralization in femurs but not in lumbar vertebrae. However, to unequivocally illustrate the region-specific regulations of leptin on the growth plate chondrocytes, we think it essential to directly measure the chondrogenic-specific markers in normal TGP and VGP chondrocytes upon leptin treatment. Therefore, in our study, we isolated and established primary chondrocyte cultures from wildtype TGP and VGP, and characterized multiple cellular and molecular events when these primary cell cultures were treated with different concentrations of leptin. Our results clearly show leptin exerts an inhibitory effect on VGP chondrogenesis, while such an effect is not observed for TGP.

In the current study, we further observed that an optimal concentration (50 ng/mL) of leptin may be required for normal TGP chondrogenesis (Fig. 4a): neither higher or lower concentrations of leptin than 50 ng/mL would further promote expression of chondrogenic marker genes *col2a* and *acan* in TGP. It is worth noting that the “optimal” concentration in our study is comparable to what has been reported for normal serum concentration of leptin [24, 25]. Therefore, the existence of optimal leptin concentration may suggest a possibility that the effect of leptin in TGP can be saturated at the physiological level of leptin. Future work should address the mode of action of leptin regarding its differential regulation in TGP and in VGP.

In this study, we established the primary chondrocyte cultures as an in vitro model to demonstrate the distinct effects of leptin on TGP and VGP. Although the findings with C57BL/6 mouse primary cultures could potentially be recapitulated in primary chondrocytes from *ob/ob* mouse, caveats should be raised against a complex scenario where defects in bone development have been established in the mutant mouse. Further studies should address the relationship between chondrocyte proliferation and the defective bone development in *ob/ob* mouse. Unless such relationship has been clarified, any results of exogenous leptin on *ob/ob* primary chondrocytes should be interpreted with cautions and with proper controls.

## Conclusions

In conclusion, leptin differentially regulates the chondrogenesis in TGP and in VGP. At the cellular level, leptin inhibits proliferation and promotes apoptosis in VGP. At the molecular level, multiple chondrogenic markers are downregulated by leptin in VGP, indicating VGP chondrogenesis is subject to the negative regulation of leptin, which is in contrast to the promotive role of leptin in the TGP chondrogenesis. In light of the critical role of cellular proliferation and apoptosis in chondrocyte terminal differentiation, the distinct functions of leptin on bone development in the vertebral and appendicular regions may have significant clinical implications. Demonstrating the mechanistic details of how leptin dictates the fate of chondrocytes in the vertebral and appendicular growth plates will assist with the understanding of the molecular pathogenesis of abnormalities in bone development, and therefore is of significant interest to clinicians.

## Abbreviations

BMC: Bone mineral content
BMD: Bone mineral density
PCNA: Proliferating cell nuclear antigen
PI: Propidium iodide
TGP: Tibial growth plate
VGP: Vertebral growth plate
